# Data-Texts in the Sciences: The Evidence-Explanation Continuum

**DOI:** 10.1007/s11191-021-00225-y

**Published:** 2021-04-28

**Authors:** Richard Duschl, Lucy Avraamidou, Nathália Helena Azevedo

**Affiliations:** 1 Caruth Institute for Engineering Education, Southern Methodist University, Dallas, TX USA; 2 Institute for Science Education and Communication, University of Groningen, Groningen, Netherlands; 3 Interunit Program in Science Education, University of São Paulo, São Paulo, Brazil

## Abstract

Grounded within current reform recommendations and built upon Giere’s views (1986, 1999) on model-based science, we propose an alternative approach to science education which we refer to as the *Evidence-Explanation (EE) Continuum*. The approach addresses conceptual, epistemological, and social domains of knowledge, and places emphasis on the *epistemological conversations* about data acquisitions and transformations in the sciences. The steps of data transformation, which we refer to as *data-texts*, we argue, unfold the processes of using evidence during knowledge building and reveal the dynamics of scientific practices. Data-texts involve (a) obtaining observations/measurements to become data; (b) selecting and interpreting data to become evidence; (c) using evidence to ascertain patterns and develop models; and (d) utilizing the patterns and models to propose and refine explanations. Throughout the transformations of the EE continuum, there are stages of transition that foster the engagement of learners in negotiations of meaning and collective construction of knowledge. A focus on the EE continuum facilitates the emergence of further insights, both by questioning the nature of the data and its multiple possibilities for change and representations and by reflecting on the nature of the explanations. The shift of emphasis to the epistemics of science holds implications for the design of learning environments that support learners in developing contemporary understandings of the nature and processes of scientific practices.

## Introduction

Faced with the COVID-19 pandemic, scientists and policymakers as well as the general public ought to consider scientific models and critically evaluate scientific evidence (Shea et al., 2020). It is no surprise then that the production of scientific research (Lees, 2020) as well as online searches for scientific information has also increased tremendously (Charlton, 2020). To live in a pandemic or any other natural disaster while immersed in the production of scientific knowledge has highlighted the challenges that world leaders, decision makers, and the general public face in critically evaluating and engaging with scientific information, especially considering the speed with which fake news and movements of scientific denialism have been rising (Ashoka, 2020; Miller, 2020).

Discussions about the impact of social/physical distancing on mental health, the types of masks, the virus survival time on different surfaces, the types of treatment for patients, epidemiological models, the possibility of a vaccine, the logistical limitations of virus containment strategies, and so many other issues exemplify the additional challenges of being immersed in coronavirus pandemic. Concurrently, global reports showcase how global scientific and mathematical literacy, which is essential for full citizen participation, is not at a desirable level (OECD, 2018). Education in general, and science education in particular, has a crucial role to play in addressing such challenges. Hence, it is important, perhaps now more than ever before, to have a critical view of the current state of affairs of science education and in light of current reform efforts. Our purpose in this conceptual paper is to examine these issues through a theoretical exploration of the role of evidence in scientific explanation construction and knowledge production. Hence, our purpose is twofold: (a) to introduce the Evidence-Explanation (EE) continuum as an alternative framework to enhance perspectives on inquiry-based science as knowledge building and refining activities; and (b) to illustrate oft overlooked key phases within the EE continuum, namely, the nuanced and situated transformations and enactments involved in negotiating the construction of evidence from data.

The manuscript consists of two main parts, one that deals with theoretical explorations and one that translates these theoretical explorations into practice by offering concrete examples of how these might look like in classroom practice. In the first part of the manuscript, we discuss the role of language in science and we introduce the concept “data-texts” in science education. Following on that, we provide a historical overview of key approaches to science education and we explain how data-texts fit within the evidence-explanation approach. Next, we describe the data-texts transformations from questions, measures, and observations to evidence and explanations.

Following these conceptual explorations, in the second part of the paper, we discuss the implications of the EE approach for curriculum design and we provide concrete examples from existing curriculum materials and classroom-based examples. We conclude with a discussion about the value and potential an evidence-based approach and an emphasis on data-text might have in reconceptualizing science education and science curriculum in times of scientific debates, challenges, and steps taken to respond to crises.

## Data-Texts in Science Education

Language is critical for creating meaning and connecting new experiences with prior knowledge (Vygotsky, 1986). In the case of the scientific disciplines, the language and practices of science can be generally characterized by model and theory building/refining processes involving evidence and explanation (Chalmers, 1999; Duschl, 1990; Kuhn, 1970). Of course, what comes to constitute and count as evidence and as an explanation are equally important epistemological considerations that complicate the growth and revision of scientific knowledge. Furthermore, the language of science is one that embraces both natural and “artificial” languages (e.g., computer languages, mathematics, symbols). Pulling together all the various and sundry ways that science both obtains and reports observations, hypotheses, measurements, models, theories, laws, and principles, among others, led Ackerman (1985) to use the term *data-texts* to characterize the growth of scientific knowledge.

Data-texts can be thought of as the multitude of representational and communication conventions used in science, technology, engineering, and mathematics to coordinate and conduct inquiries and investigations (Ackerman, 1985). A dilemma that Ackerman posed then for the sciences, and one we believe importantly extends to current matters of science education, is that the language and reasoning conventions used to “talk” about data-texts have not kept pace with the rapid generation of relational mathematics found in data analytics and the data-texts. In 2016, the National Science Foundations launched the INCLUDES program with the aim of transforming STEM education and career pathways. One of the 10 Big Ideas proposed is “Harnessing Data for the 21st Century Science and Engineering.” Simply stated, our representations of the practices and languages of science have not kept up with our scientific theoretical knowledge nor with the tools and technologies now being deployed. In attempting to address this problem, we propose an emphasis be placed on the conversations about science, or, in Ackerman’s terms, the data-texts in science education.

Central in these conversations about science is scientific reasoning. Scientific reasoning by definition involves both conceptual understanding and inquiry skills (Osborne, 2018; Zimmerman, 2005). As Zembal-Saul et al. (2013) argued, “not only should students understand and be able to apply scientific ideas to explain natural phenomena but they also should be able to generate and evaluate scientific evidence, construct and debate evidence-based explanations, and participate productively in a community of science learners” (p. 17). This view is in line with Hardy, Dixon, and Hsi’s approach (2020) that “data should be considered to be actively produced, rather than passively collected” (p. 1). This perspective represents a paradigm shift in line with what we are advocating. The idea of data-texts goes beyond the passive collection of data and implies the critical evaluation of its origin and transformations. Although, the idealized view of data ready-to-discover in nature has been addressed previously by Latour and Woolgar (1986); for example, the absence of any epistemic agency that goes through the entire process of data collection and manipulation is still missing in school inquiry practices (Duncan et al., 2018; McNeill & Berland, 2017; Stroupe, 2015).

In a recently published article on styles of scientific reasoning, Osborne (2018) reinforced the idea that reasoning in science is dependent on three dimensions of knowledge: content, procedural, and epistemic. Based on the work of Crombie (1994), Osborne argued how these three components appear in six types of scientific reasoning, namely, mathematical deduction, experimental exploration, hypothetical modeling, categorization and classification, probabilistic reasoning, and historical-based evolutionary reasoning. These practices feature centrally in our proposition for an emphasis on data-texts.

In what follows, we will argue that a missing dynamic in science education is the all-important dialectic, what we call *conversations* about science, between measurement/observation and explanation, which focuses on the processes of data transformations. Cognitive psychological, social psychological, and epistemological perspectives about learning can be used as frameworks to better understand the *conversations* about science. These conversations, essentially, reveal the processes of scientific knowledge construction and unfold the nature of scientific and epistemic practice. Our claim is that a focus on the *data-texts* conversations surrounding the acquisition of data and then the subsequent transformations of data to evidence, evidence to models, and models to explanations enhances the teaching and learning of and about science.

## The Evidence-Explanation Approach to Science

Traditional approaches to science education can be classified into two different continuums. The first is the content-process (CP) continuum approach, which focuses on teaching what we know through the lore of the scientific method. The other one is the discovery-inquiry (DI) continuum approach, which focuses on extracting patterns from interactions with nature (Duschl, 2008). The CP approach over the past 40 years has been giving ground slowly to the DI approach as our understanding of learning processes and of scientific reasoning emerged. Discovery learning has been a hallmark of science programs since the 1960s. School science programs have long been committed to discovery/inquiry teaching methods in which learners manipulate materials, conduct observations, take measurements, or otherwise participate in activities intended to demonstrate or reveal science concepts, principles, and laws (Millar & Driver, 1987). Active learning methodologies, nowadays widespread as a slogan in schools and curricula around the world, sometimes take this perspective as an inspiration, but often end up focusing only on hands-on and not minds on.

In addition to CP and DI, there is a third approach to science education, the *Evidence-Explanation* (EE) approach that emphasizes the data-texts of science. EE helps bridge the gap between authentic inquiry and DI inquiry tasks in school science. Our position, similar to others’ (e.g., McNeill & Berland, 2017), is that the EE approach can marshal the cognitive and the epistemological beliefs buried in CP and DI frameworks. What makes the EE approach different from the traditional approaches to science education is the focus on the transformations (and their nuances), which occur throughout the EE continuum. This focus lies in the emphasis on the *epistemological conversations* about science.

Engaging in the construction of scientific arguments as a way of teaching and learning science has been emphasized by a number of researchers over the last couple of decades (e.g., Driver et al., 2000; Fisher et al., 2018; Kuhn, 1993; Newton et al., 1999; Sandoval et al., 2019; Zembal-Saul, 2009). As Jimenez-Aleixandre et al. (2000) argued, “argumentation is particularly relevant in science education since a goal of scientific inquiry is the generation and justification of knowledge claims, beliefs and actions taken to understand nature” (p. 758). Students have to understand the rational basis for their actions, and for this to occur they have to work their own ways through issues “until they arrive at a consistent, acceptable position which can be defended persuasively and which takes other points of view into consideration” (Bourne and Eisenberg, as cited in Geddis, 1991, p. 11). A recent empirical study showed that the effectiveness of the argumentation depends on the assimilation of the argument idea by the students, which can occur both at the conceptual and methodological levels (Sandoval et al., 2019). Beyond this empirical evidence, we argue that such a vision is aligned with the idea that connecting discursive practices with the construction of collective meanings promotes productive argumentation in science classes.

Moreover, engaging in thinking and learning science in terms of argument discourse supports gaining an understanding of how scientists conduct their work (National Research Council, 2000, 2011), by bringing the scientific and epistemic practices conducted by students closer to the ones conducted by scientists. Central to the discourse of scientists is the development of scientific claims and theories, which are challenged and progressed through dispute, conflict, and paradigm change in the public domain (Driver et al., 2000). When students are provided with opportunities to construct arguments, gather evidence to support them, and communicate them to their peers, they are experiencing the process and culture of science in an analogous way as scientists do. Driver et al. (2000) referred to this process as enculturation into science where students not only hear explanations being given to them by experts but they also practice using the ideas themselves and develop an understanding of scientific practices and ways of thinking as scientists do. An important element of this process of enculturation into science through argumentation, we claim, is the conversations *in* and *about* science.

The call for conversations *in* and *about* science represents an acknowledgment of the value and importance of representation, communication, and evaluation in science learning. We use the term *conversations* in a very broad sense to include, among other ideas, argumentation, debate, modeling, drawing, writing, and other genres of sharing language. Such an expanded repertoire helps us to consider an important domain of research in science learning contexts, namely, how to mediate the learning experiences. The position advanced by Schauble, Leinhardt, and Martin (1997), and adopted here, is that such learning mediations should focus on promoting talk, activity structures, signs and symbol systems, or collectively what we will call *conversations.* For science learning, the conversations, which we refer to as data-texts and explored further in the following section, should mediate the transitions from evidence to explanations and, thereby, unfold the nature of the processes of scientific practice.

## Data-Texts Transformations

*Data-texts* are an element of scientific practice, and are used to refer to the process of data transformation in science from observations to explanations—a journey of both comprehension and articulation, which moves from the “what” to the “whys” and “hows” of phenomenon. That is, data-texts are a collective system of complex transformation processes through which raw data in the form of measures and observations are obtained and then examined to become the data that is used, e.g., evidence. In turn, the evidence is examined to generate scientific models and explanations.

According to Gott and Duggan (2003), the weight of evidence is associated with the way the data were obtained. In certain fields (e.g., medicine, pharmacy, and health sciences in general), evidence discussions are more situated, mainly because of its role in decision-making. In these sciences, systematic reviews are conducted in the effort to synthesize evidence from treatments or diagnoses, for example, to inform public health policies. This leads to the idea of a hierarchy of evidence which is not recent, nor is it novel in the construction of knowledge (e.g., Ackoff, 1989). A hierarchical view of the evidence considers that the evidence of a study will vary according to its inherent characteristics (e.g., observation, near-experiments, experiments, systematic reviews). On this issue, Boyd’s work (2018) provides some clarification regarding the role of context in the epistemic utility of the evidence.

To accumulate, evidence must outlive its original context. To be used jointly, differently sourced evidence must be amenable to the same context. To constrain competing theories, the same evidence must be adaptable to different contexts. (p. 405)

The origins of the data and their contextual dependence enable the accumulation and fusion of evidence. In this work, Boyd points out the importance of looking closely at the processes that generate and treat data, which implies the reflection about the instruments, as also discussed previously by Ackerman (1985). Our position is that evidence determination is based on what kinds of data are selected, which are determined by adopted guiding conceptions (Schwab, 1962) of theoretical, methodological, and axiological commitments.

Our view of scientific explanations is that they provide an understanding of the world. As cited in Trout (2002) and in agreement with Brewer, Chinn, and Samarapungavan’s analysis (1998), scientific explanations are no different than everyday explanations that provide a conceptual framework of a phenomenon that leads to a feeling of understanding in the reader-hearer. However, these explanations would be based on different forms of scientific reasoning. The epistemological conversations would be a way to foster the high-order analytical skills necessary for scientific reasoning. Discussions around data-texts can be an opportunity to highlight two dimensions that Klahr et al. (2011) pointed out as fundamental to scientific reasoning: content and processes. Expanding this perspective, Osborne (2018) argued that it is also necessary to go further by offering “means of identifying both the distinctive forms of reasoning and the knowledge required for their undertaking” (p. 172). According to Osborne, placing scientific reasoning at the center of science curricula demands a recognition that the styles of reasoning (and their products) are interdependent and guided by distinctive ontic, procedural, and epistemic constructs.

**Fig. 1:**
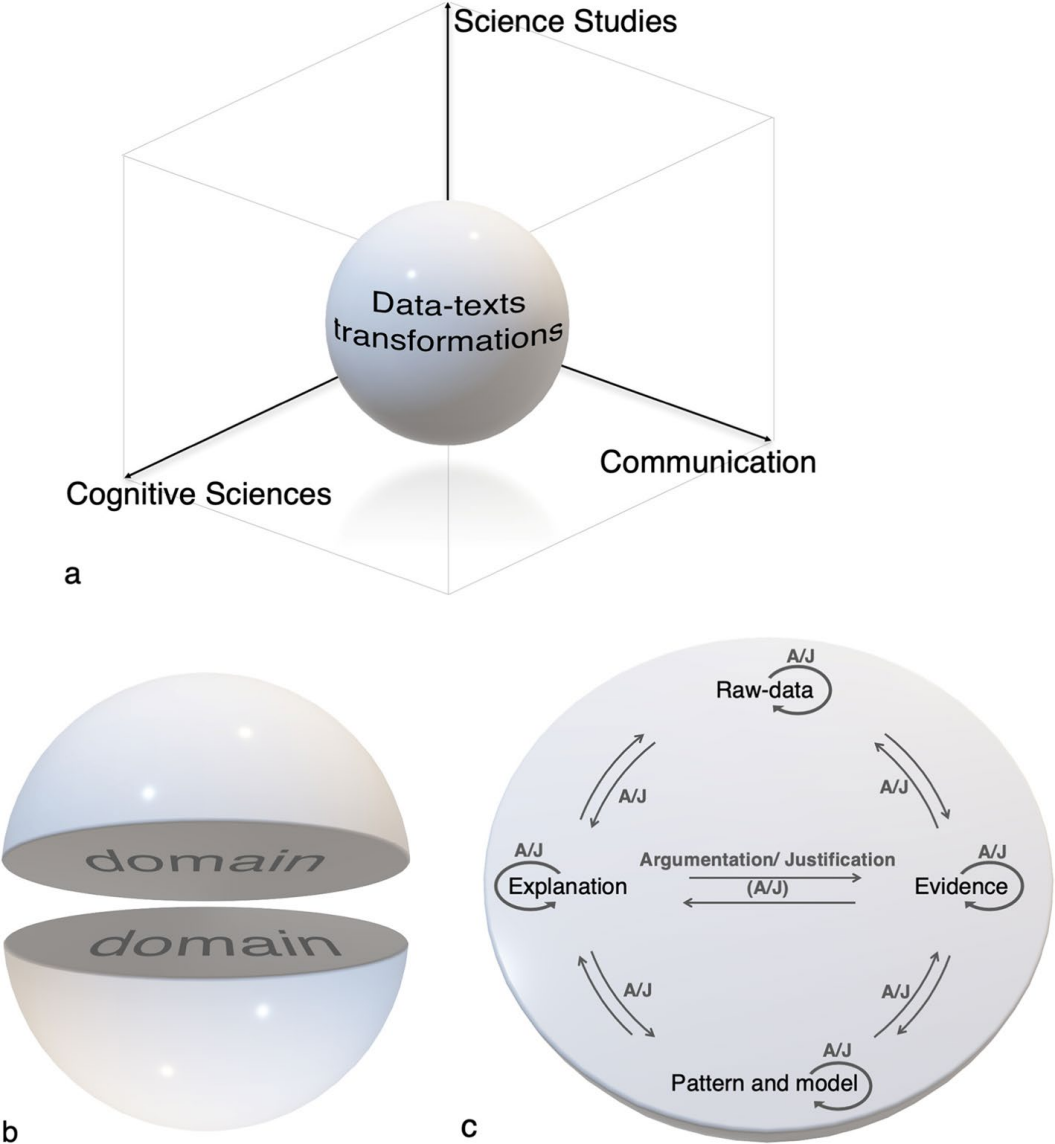
The three levels of representation to understand the *data-texts transformations*

A visual formalization of data-texts transformations is presented in Fig. 1, in which we highlight the layers of interpretation of this concept. In Fig. 1a, we point out the theoretical dimensions that support the data-texts transformations, placed at the center of the theoretical space comprising the projected area of the three axes. The three theoretical axes that support this concept (Table 1) include Cognitive Science, Science Studies, and Communication. The interdependence of the axes is consistent with the contributions of Ackerman’s and Giere’s ideas.

**Table 1:** Theoretical spaces for data-texts transformations

Theoretical axes	Description
Science studies	Focus is on the theoretical contexts and/or the disciplinary domains in which data-texts emerge
Cognitive sciences	Focus is on the data-text transformations that take place when mobilizing cognitive and metacognitive processes related to learning and perception
Communication	Focus is on the way data-texts are expressed through inscriptions: e.g., diagrams, maps, photographs, data tables, writing texts, graphical representations

In Fig. 1b, we go further to represent the idea of data-text transformation, emphasizing that this transformative process emerges from a domain/context (which comprises the internal volume of the sphere). In Fig. 1c, we present the process of data-texts transformations itself, which occurs immersed in a context domain-dependent and, therefore, in the internal volume of the sphere. The data-texts transformations emerge from epistemological conversations and are consequently not linear, but dynamic, occurring in moments of collective production of knowledge, with multiple reflection points, evaluations, and feedback loops. Data-texts are essentially a collective system of complex transformation processes through which raw data are obtained and then examined to become evidence, evidence and patterns of evidence are used to develop models, and the models and patterns are employed to propose explanations. The arrows (junctions) in Fig. 1c indicate the transformations of the data-texts and the points for decision-making based on argumentation and justification (A/J), which are fundamental actions in the collective construction of knowledge.

The concept of data-texts helps to reveal the growth and development of scientific knowledge; which is mediated through communication of the discursive dynamics of “data to model” transformations itself. As we argued earlier, data-texts can be considered the multiplicity of representation and communication conventions used in sciences to conduct investigations. Data transformations is a process that does not happen in isolation; instead, it is embedded in social, epistemic, and cognitive consolidation processes that are situated within sociocultural contexts and specific knowledge building learning environments. Such processes and interactions with the cognitive processes and particularly with what Giere (2002) called distributed cognition:

A situation in which one or more individuals reach a cognitive outcome either by combining individual knowledge not initially shared with the others or by interacting with artifacts organized in an appropriate way, or both. (p. 641)

Distributed cognition, we claim, is appropriate to frame the social nature of data-texts because it emphasizes the social system of people’s specialized knowledge and the instruments’ capabilities. The term *collective* is used here not as a framework for the social accounts of science but as one to help us better understand how the cognitive system of data transformation works within the social nature of data-texts. As Giere (2002) puts it, “to know how a cognitive system works one has to know about the culture and social organization as well as about the capabilities of the people and the artifacts” (p. 641). We describe data-texts as complex because it is not a straightforward, clear-cut process; instead, it entails errors, and at times missing, conflicting, and anomalous data.

Thus, data-texts resemble a sort of circular motion because there is no end to possible streams of transformation, as explanations undergo change in light of new evidence and/or alternative interpretations of existing evidence. Data-texts also change as the existing theories that people use to make sense out of observations and interpret evidence to construct explanations change as well (Fig. 1). Discussing the relationships between observations and theories, Chalmers (1999) argued that,

The recording of observable facts requires more than the reception of the stimuli, in the form of light rays that impinge on the eye. It requires the knowledge of the appropriate conceptual scheme and how to apply it (p. 12).

In agreement with this view, we maintain that observations are theory-laden as we view science being theory-dependent. Therefore, we consider existing knowledge, understandings, and theories to serve as a baseline in the data transformation processes, as they influence the ways in which people filter, prioritize, make meaning out, and interpret observations to be used as evidence. As Hanson (1971) pointed, “the conceptual shape of one’s theories, the posture and stature of one’s presuppositions, determine where observations have to be cleaned up – where they should be realigned and reprocessed effectively to be plugged into a science’s theoretical framework, its structure for intelligibility” (p. 7).

Data-texts then help generate explanations, yet they remain dependent to the origin of the phenomenon and are interconnected to existing observations and experiences as they incorporate new observations and interpretations. We argue that data progress to explanations through a series of epistemic and social conversations that involve interpretations of evidence, construction of arguments, and negotiation and communication of arguments. These conversational processes resemble in a sense the work of scientists and research groups who engage in intellectual dialogs as they share their work and become informed about other scientists’ work through collaboration, peer review, presentations in public forums, and publication of their work. Such activities illustrate the social nature of knowledge. Longino (1990, 2002) argued that theories are influenced by social and cultural values. Nevertheless, scientific inquiry, according to Longino, can maintain its objectivity by understanding it as a social rather than an individual process. For Longino, scientific activity is a collaborative human activity. Beyond considering the scientific activity as a practice, Longino (2002) also argued about the importance of discursive interactions for knowledge production.

Data-texts also build on theoretical foundations from cognitive science focusing on cognitive structures, processes, and models (Fig. 1). Our vision of data-texts is influenced by perspectives from the *cognitive-historical* method of studying science proposed by Nersessian (2003). This line of work aims at identifying various cognitive practices that are employed in scientific cognition and developing explanatory accounts of the generativity of the practices.

Cognitive-historical analysis creates accounts of the nature and development of science that are informed by studies of historical and contemporary scientific practices, and cognitive science investigations of aspects of human cognition pertinent to these practices. The historical dimension of the method is used to uncover the practices scientists employ…the cognitive dimension factors into the analysis how human cognitive capacities and limitations could produce and constraint the practices of scientist. (p. 135)

Thus, the aim of the cognitive-historical approach to science is one of illustrating the “historized epistemology” of science and reconstructing scientific thinking by means of cognitive theories. The role of “historicity” of epistemology is important in framing data-texts because it suggests that the construction of scientific explanations is a *contextualized revolutionary* or *evolutionary process* rather than an evolutionary, sudden change detached from specific contexts. Such a revolutionary process, which is situated within specific cognitive and social contexts, is consistent, we maintain, with data-texts (Fig. 1b). In fact, it is upon this cognitive-historical method that the account of model-based reasoning derives, which is used to refer to a form of knowledge organization, and figures central in data-texts. As Nersessian explained, “what is special about models is that they are designed so that elements of the model can be identified with features of the real world, which makes it possible to use models to *represent* aspects of the world” (p. 8).

We further ground data-texts to Giere’s (1986) view that models serve as representation of the world and theories are made up of as *families of models.* According to Giere (1986), “understanding science is primarily a matter of understanding the cognitive processes of scientists involved in doing science” (p. 322). The slogan of this cognitive approach to science, as Giere (1986) puts it, might be: “the task is not to justify science, but to *explain* it” (p. 324). Over the last few decades, deliberations about the nature of science have indicated that a well-informed view of science depends on both the social and cognitive dynamics when developing scientific knowledge.

The shifting focus that we are proposing is consistent with approaches that are committed to teaching and learning about the nature of science, such as the family resemblance approach (FRA) presented by Erduran and Dagher (2014). The model describes science “as a cognitive-epistemic and social-institutional system” (p.155), and this idea is expressed in the three-circle concentric model named FRA wheel. The inner-circle concerns scientific practices; the next circle concerns the values, objectives, and sociocultural practices of science; and the external circle concerns the institutional and sociocultural factors of science. The model represents science as a dynamic and holistic system that is under the influence of different sources (Kaya & Erduran, 2016). One could argue that *data-texts transformations* are essentially at the center of the FRA wheel model, where aims and values, practices, knowledge and methods, and methodological rules are present in the collective scientific actions and practices. The *epistemological conversations* make it possible to highlight the multiple relationships that emerge from the alignment of components when the concentric circles are rotated (connecting, for example, practices with both the social certification and dissemination component and the social organizations and interactions). In this way, we believe that the EE continuum dialogs with the approach, in the sense of contributing to the construction of better-informed visions of science, when it systematizes epistemic practices within a continuum and fosters epistemological conversations throughout the process of scientific making.

## Implications for Curriculum Design

**Fig. 2:**
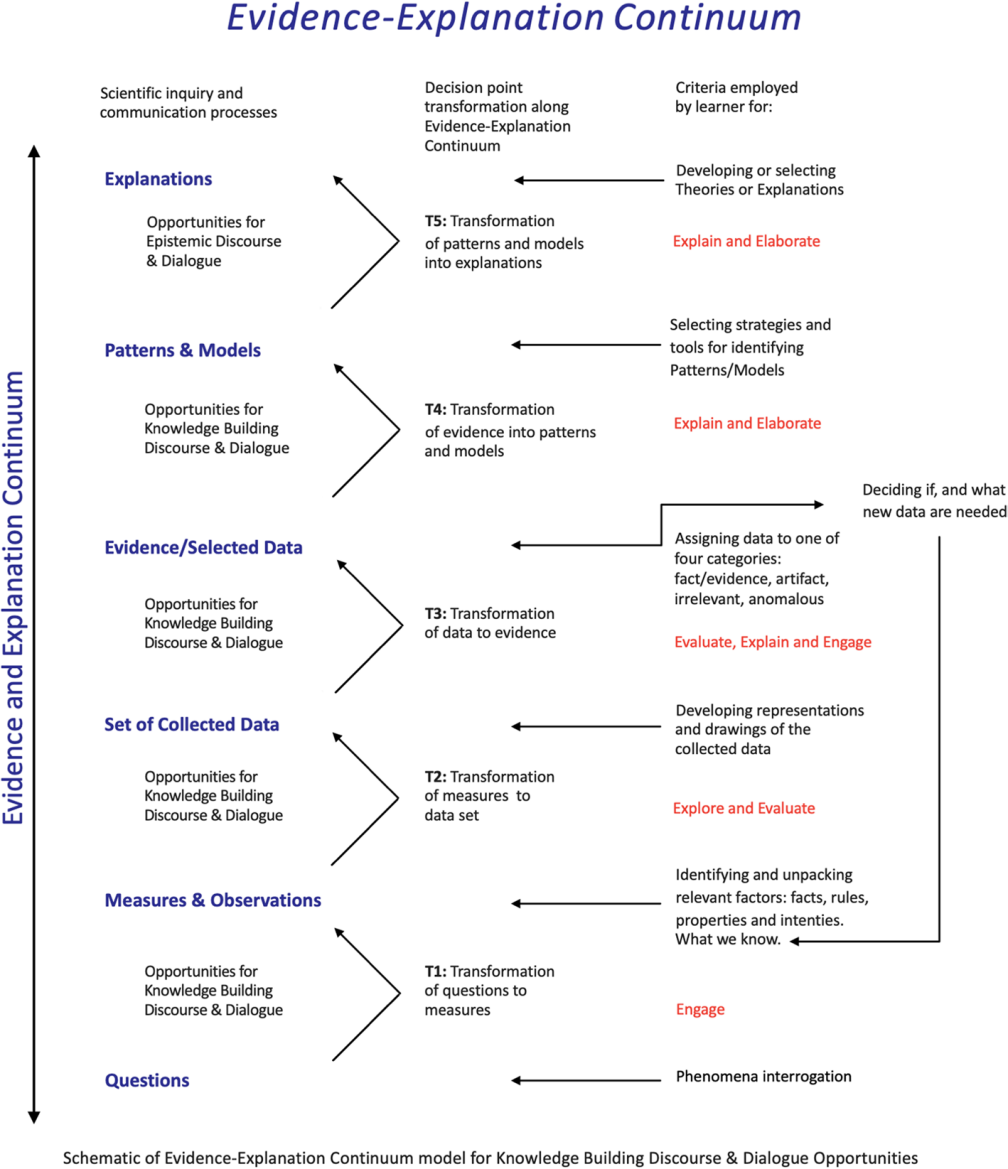
Expanded schematic model of the Evidence-Explanation continuum for knowledge building discourse and dialog opportunities

The appeal to adopting the EE continuum as a framework for guiding the design of science education curriculum, instruction, and assessment models is that it seeks to work out and provide instructional guidance for the knowledge building and refining dynamics presented in Fig. 2. The EE continuum framework, previously presented in Duschl (2000), Kelly and Duschl (2002), and Duschl and Grandy (2008) at three stage process, is based on the premise that scientific investigation is a process of knowledge transformation. The expanded approach to this framework proposed in this paper includes two additional transformations: *transformation of questions to measure/observations* and *transformation of measures/observations to data sets*, presenting the multiple possibilities for epistemic conversations in science lessons. Throughout the EE continuum, students and teachers need to engage in a diverse range of decision-making, engaging in a teaching and learning environment that fosters the use of inscriptions to communicate data and information. Accordingly, the transformative process of the EE continuum does not only mobilize a diverse range of epistemic practices but also reflects, in a broad and holistic way, the 5E’s (engage, explore, explain, extend, and evaluate) required in the pedagogical practice of inquiry (Bybee, 2015).

The EE continuum materializes how cognitive structures and epistemic processes guide judgments about data-texts. In science activities, the data-texts transformations are guided by the inquiry process, deriving from questions, measures, and observations (T1 and T2 in Fig. 2). It does so by formatting into the instructional sequence select junctures of reasoning, what we call *data-texts transformations* (as shown in Fig. 1). At each of these junctures or transformations, instruction pauses to allow students to make and report judgments for building collective knowledge. Then, students are encouraged to engage in rhetoric/argument, representation/communication, and modeling/theorizing practices. The critical transformations or judgments in the EE continuum include:

- Collecting data through observations and investigations
- Selecting data to become evidence
- Evaluating evidence
- Using evidence, patterns of evidence and models
- Employing the models and patterns to propose explanations

These decisions and judgments are critical entities, we argue, for explicitly teaching students about inquiry and the nature of science (Duschl & Grandy, 2012; Duschl et al., 1999). How raw data are selected and analyzed to be evidence and how evidence is selected, evaluated, and analyzed to generate scientific explanations are important “transitional” steps in doing science. Throughout the transformations of the EE continuum, there are stages of transition that foster the engagement of learners in negotiations of meaning and collective construction of knowledge (e.g., T3, T4, and T5). Each transition involves data-texts (materialized in multiple inscriptions) and making epistemic judgments about “what counts.” As Duschl et al. (1999) explained:

The complex relationship between evidence and explanation in science warrants an examination of changes or boundary adjustments in three kinds of criteria children have to relate evidence to explanation: a) criteria for assigning data to one of four categories: fact, artifact, irrelevant or anomalous; b) criteria for identifying patterns/models in selected data; and c) criteria for theories or explanations created to account for the patterns/models. (p. 532)

The central role that progressive decision-making about data becoming evidence, evidence becoming models, and models becoming explanations has in doing science suggests there is an advantage to merging together cognitive frameworks of philosophy, psychology, and pedagogy. According to Sandoval et al. (2014),

Coordinating claims and evidence requires a number of reasoning practices to evaluate the strength of evidence, critique methods and other factors upon which evidence evaluation rests, evaluate sources and potential biases, and so on. (p. 140)

Lehrer et al. (2001) argued that the role of experimentation in science classrooms should abandon the disembodied method of knowledge verification. This is consistent with Giere’s argument to move the goal of the philosophy of science from justification to understanding. The position is to think of science as model building and revision, and in the case of students doing investigations to adopt a conception of experiments as models and experimentation as modeling.

## Data-Texts Transformations in the Evidence-Explanation Continuum in the Classroom

In the previous sections, we emphasized the importance of data-texts as a knowledge building dynamic. However, it is also important to highlight and explain how the *epistemological conversations* that support a switch to the EE continuum could help students to build ideas that are better informed about what constitutes evidence. Research programs are currently underway to analyze the nature of evidence in the context of science education (e.g., Duncan et al., 2018; Greene et al., 2016; Krist et al., 2019; McNeill & Berland, 2017). For example, McNeill and Berland (2017) reflected on the poor understanding of the role of evidence in school scientific activities. They then proposed design heuristics as implementation possibilities for science education that include the following: (a) a focus on EE continuum; (b) the use of evidence that is based on phenomena; and (c) a look at the evidence that is applied in the arguments.

Despite the fact that research in science education has focused on how students engage in activities that require the use of evidence (either using evidence to argue, build models, or assess its quality), little attention has been devoted to how students deal with the production and development of evidence itself (Duncan et al., 2018). A focus on the EE continuum may foster teaching and learning environments in which questions about what counts as evidence are more likely to emerge. This is relevant because in the sciences, in general, some evidence may have more strength than others (which may, for instance, depend on the quality of the measurements, the sample size, or the credibility of the information sources). The consciousness of this might contribute to distinguishing science from pseudoscience, for example.

The essence of what we are proposing can be exemplified with a few questions addressed in epistemological conversations related to asking questions of the data and through problematization of evidence, such as the following: How were the data collected? Why were the data collected in this way? Why was one technology chosen over another? For how long was the data collected? How were the analyses validated? Why were no further measures taken? What statistical test was used? Questions of this type (focused on the initial transformations of the EE continuum) also make it possible to reflect on the level of collective knowledge we have about a topic since lower levels of evidence can support the initial comprehension of patterns (e.g., when we still do not have precise explanations for the phenomenon observed). This is a point that would allow exploring pedagogically, for example, the situated (historical and theoretical) nature of the data and explanations, and theoretically illustrates a possibility for reflection throughout the EE continuum.

Aligned with this approach, Duncan et al. (2018) proposed a five-dimensional framework to help build a more sophisticated and authentic view of the evidence. Such dimensions, which include analysis, evaluation, interpretation, integration, and use of evidence, involve several epistemic practices. We maintain that such framework dialogs are consistent with the EE continuum since the boundaries of their continuum depend on successive and iterative dialogic steps that embrace a deep understanding and use of robust evidence.

The question then becomes one if everyday life interactions are based on such deep understanding and use of robust evidence. The answer is no. For instance, it is common to use cases of anecdotal evidence in everyday life to support decisions, even if this type of evidence is considered dubious in itself. For this very reason, the idea of evidence, assuming that this idea is at the heart of the scientific practice, is a knowledge that can be considered what Young (2008) calls powerful knowledge. This is because the concept of evidence, as well as its limits and biases, is a knowledge that allows understanding the world beyond the walls of the school and that can be used in new contexts. A focus on evidence and its hierarchies can help learners build better-informed ideas about how to weigh the evidence (strong, weak, contradictory, etc.). The knowledge about evidence could help them build a repertory about the quality and validity of available information around them, especially as the level of evidence is rarely explicit or easy to access. This paradigm change at the individual level about the nature of the evidence involves understanding that data will be transformed into evidence when meaning is assigned to it since the evidence is associated with the construction of a hypothesis and an argument that is desired to be explored.

Science proficiency involves both generating and evaluating the role of evidence in the construction of models and explanations, which is associated with the nature of scientific knowledge. When we abandon the DI approach (mostly based on the experimental studies with the deductive hypothetical method), we create space to broaden the *conversations in* and *about* science, allowing to focus on aspects especially related to biases in methodological choices. In this context, the EE continuum can support the design and implementation of learning progressions since it helps identify and define key learning points and skills to be developed in science classes (Duschl, 2019). It is worth considering that learning progressions do not necessarily represent fixed or linear journeys (Pierson et al., 2017). There exists evidence in the knowledge base that both context and instructional scaffolding can impact on how students engage in learning progressions (Berland et al., 2015; Pierson et al., 2017). Thus, structures such as the EE continuum can be a valuable planning and evaluation tool.

## What Do Data-Texts Look Like in Practice: a Classroom-Based Example

To exemplify the potential of data-texts transformations through epistemological conversations, we selected as a case study the work of Lehrer and Schauble (2002, 2004), also presented in National Research Council (2008). The authors assessed 5th-grade students’ understanding of the natural variation in the growth of 63 Wisconsin Fast Plants in an experimental design conducted in the school context. Over 40 days, students generated, evaluated, and reviewed a variety of data. In discussing changes in the distributions of the measures they collected, they interpreted what the “data forms” said about changes in plants. What makes this activity a good example for us is that, in addition to being an example of learning progression, it explores important dimensions of data-texts transformations, such as the need for theoretical recognition of the theme (the context), the transformation of the data to the construction of explanations, and the interdependence of the data with the nature of the investigation (as shown by the theoretical framework represented in Fig. 1).

The students’ activity involved reporting in small groups the plant growth data. The first decision level was precisely to define what to measure, how to measure, and what is or is not a good measure of growth (T1 and T2, Fig. 2). Decisions constantly used the justification and argumentation, mobilizing the three axes of data-texts transformations (Fig. 1a). After data collection, the groups needed to show what was the typical growth situation of a plant on specific days, which allowed, again, new conversations about the nature of the data, since there is no uniform height in the plants studied (T2 and T3, Fig. 2). The groups’ responses, which included, for example, values within an interchangeable range, allowed for another level of discussion highlighting the relationships between evidence and patterns (T4, Fig. 2). At this point, it was possible to create spaces for thinking about the methodological limitations that each group faced. The decisions made by the students on how to carry out and record the measurements could impact the answer of each group and, for this reason, led to reviews by the students (exploring and revisiting the transformations that take place throughout the EE continuum).

At another moment in the activity, the students had to predict what could happen if they followed the growth of the plants again. According to the authors, the “aim was to invoke an image of a (random) repeated process, with a sampling distribution as a way of characterizing the likely outcomes of the repetitions. By drawing samples from urns, students explored different sampling models (without and with replacement) and effects of sample size and number of trials” (p. 644), emphasizing that at the core of this question is not just the discussion about the character (and hierarchy) of the evidence, but mainly the complex relationship between evidences and explanations, mediated by inference. Finally, after evaluating the frequency distribution of plant heights over time, students were instructed to reassess their initial predictions about light and nutrients, essentially based on the data. This re-evaluation mobilized the different directions of the epistemological conversations mediated by argumentation, as described in Fig. 1c, and allows reviewing the practices and transformations that occurred throughout the EE continuum (Fig. 2).

This case study highlights the role of communication via inscriptions (exemplified by the different diagrams and graphics prepared by students), which is evidenced through epistemological conversations, mobilizing and clarifying the mutual importance of the three axes of data-text transformations (Fig. 1a). The idea of data and evidence is contrasted with the patterns and models when students need to make predictions (Fig. 1c), since students were asked how the graphs they prepared and the data they collected support the explanations they provided to the questions. The many decision-making points, which include how the data were collected, organized, and presented, helped students to support interpretations of the effects of adding light and nutrients. One of the learnings that can be associated with this outcome is the recognition that the data and their interpretation depend on a context, be it theoretical (and therefore also methodological) or sociocultural.

We chose this example because it lends itself to the opportunity to discuss how students create and interpret data representations, as well as to emphasize how educators need to structure and provide such learning opportunities (Duschl, 2019). The conversations in this context may advance, for example, our understanding of whether there is sufficient evidence to support a conclusion and what kind of additional data would be required for the construction of an explanation. This dialog is important because through it a recognition that the same pattern, model, or phenomenon may have multiple interpretations, which may or may not lead to alternative explanations, might emerge. In this context, epistemological conversations can offer the means to recognize the value of explanations in science, which both help to foster new questioning and can reveal limits to the understanding of available evidence.

Although several studies have demonstrated results that indicate student engagement and an improvement in science learning throughout inquiry (e.g., Fang, 2020; Kruit et al., 2018; Minner et al., 2010), there are still rare initiatives that organically permeate the whole EE continuum. Thinking about using the transformations indicated in the EE continuum for curriculum planning (such as the design of learning progressions and assessments), we organized Table 2. Table 2 goes beyond the didactic example that we explored in this section and indicates some questions which can help teachers, researchers, and curriculum designers visualize and materialize how the different transformations take place throughout the EE continuum (Fig. 2). By keeping the proposed questions in mind, it is possible (i) to think about opportunities for discussion to engage students in epistemic practices that shape scientific investigations, (ii) to think about possibilities for creating didactic activities, and (iii) to think about ways to analyze research data.

**Table 2:** Examples of questions and studies that illustrate how transformations along the *EE continuum* can take place in the classroom

Transformation along *EE continuum*	Examples of questions to foster epistemological conversations	Examples of studies whose data help to understand how transformation can take place in the classroom
T1 (questions → measures)	- Is it possible to obtain measures to answer the research question? - What measures can help to answer the question? - What measures can be collected in our context? - How could the experiment be made? What are the variables and constants in the experiment? - What is already known about the subject which could help to improve the research question and to think about the possibilities of measures?	Lehrer and Schauble (2002, 2004) Chin and Kayalvizhi (2002) Bjønness and Kolstø (2015)
T2 (measures → data set)	- How will the data be collected? What instruments and/or techniques will be used? - Which data will be collected, organized, and registered? - What are the biases of the measures adopted as a reference? - What will be considered as data? Which data will be discarded? - What criteria will be used to distinguish fact, artifact, anomalous data, and irrelevant data? - How many data will be collected?	Lehrer and Schauble (2002, 2004) Wu and Krajcik (2006) Bjønness and Kolstø (2015) Crujeiras-Pérez and Jiménez-Aleixandre (2017a) Crujeiras-Pérez and Jiménez-Aleixandre (2017b)
T3 (data → evidence)	- Are there any other data that could be more informative? - How will the data be analyzed (statistically, graphically, qualitatively)? - Should the data be transformed into charts? - Should the data be transformed into an index? What is the best way to do this? - Can the data be organized in a diagram? - Can the data be organized to explain a pre-existing hypothesis/model/pattern? - What are the results of the data analysis?	Lehrer and Schauble (2002, 2004) Wu and Krajcik (2006) Bjønness and Kolstø (2015) Crujeiras-Pérez and Jiménez-Aleixandre (2017a) Bravo-Torija and Jiménez-Aleixandre (2017) Crujeiras-Pérez and Jiménez-Aleixandre (2017b)
T4 (evidence→ patterns and models)	- What is the best type of graph to represent the pattern/model? - What are other possible forms of representation/ inscriptions to illustrate the pattern/model?	Lehrer and Schauble (2002, 2004) Wu and Krajcik (2006) Prain and Tytler (2012) Bjønness and Kolstø (2015) Bravo-Torija and Jiménez-Aleixandre (2017) Crujeiras-Pérez and Jiménez-Aleixandre (2017b)
T5 (patterns and models → explanations)	- What is the possible explanation/what is the theory for the model/pattern found? - Does the model/pattern found explain the phenomenon investigated (which was in the initial question)? - Are there other explanations for the pattern/model? - How to justify the conclusions? - What can be concluded from the evidence? - Is there a consensus (collectively built) for the proposed explanation? - What are the implications of this explanation? Is it possible to generalize it?	Lehrer and Schauble (2002, 2004) Wu and Krajcik (2006) Prain and Tytler (2012) Bjønness and Kolstø (2015) Bravo-Torija and Jiménez-Aleixandre (2017)
T5 → T1	- Are there alternative explanations for the question? - What questions could help corroborate alternative hypotheses to answer the research question? - What else could have been done to answer the question? - Why doesn’t the explanation answer the question? - What other questions emerged from the investigation?	Bjønness and Kolstø (2015)

We agree with Manz et al. (2020) that, in general, the vision of science as a practice (which involves the different aspects of inquiry) is not a process visible to learners. Hence, by carrying out an operationalization, the EE continuum also aligns with the idea of planning and carrying out investigations. This is because, by highlighting actions that permeate and compose its continuum, there is an opportunity to facilitate both planning and action in the classroom, enhancing the visualization of processes associated with scientific investigations and knowledge production. Accordingly, opportunities are provided for students to take measurements, explore their meanings by sharing the significance of the data, consider and decide through an epistemic community what counts or not as evidence, etc.

## Concluding Remarks and Implications

Even though there is a wealth of studies on argumentation and sense-making in science education, and an increasing number of papers advocating for the explicit teaching of the nature of science (Azevedo & Scarpa, 2018), the significance of developing reasoning skills associated with the evaluation of evidence and the construction of evidence-based explanations has not received enough attention in the field of science education (Sandoval et al., 2014). Chin and Kayalvizhi (2002) argued that “the ability to reason well about complex models of data is essential not only for scientists but for nonscientists as well” (p. 213). Through an examination of the data-texts and their role in model-based science, we made claims about placing emphasis on the epistemic practices that are connected to processes of scientific reasoning. In particular, we argued about the significance of the *epistemological conversations* about data transformations in science. We refer to these transformations as *data-texts* and we build upon this concept to introduce a dialogical approach into science education, where the EE continuum is central. As Kelly and Duschl (2002), argued:

The complex relationship between evidence and explanation in science, a relationship that harbors conceptual, epistemological and social discourse dynamics, warrants a systemic examination of understanding the development of the criteria learners employ to relate evidence to explanations (p. 33)

Thus, we have emphasized the critical transformations in the EE continuum: selecting data to become evidence, using evidence to develop models, and employing models and data patterns to build and refine explanations. An emphasis on the data-texts illustrates the social nature of knowledge and learning, and the role of language and discourse in the construction of scientific knowledge. The shift in focus to the EE continuum allows exploring aspects of the nature of science such as (a) the transformation of data into evidence; (b) the situated context of observations and theories; (c) the role of theories and previous knowledge in data collection; (d) the variety of methods in science; and (e) the role of models and evidence for the transformation of knowledge.

It should also be considered that, in recent years, science education research has indicated that students can have a considerable improvement in learning scientific concepts when opportunities are provided to engage in scientific investigations. Finally, at the heart of all this discussion is the possibility for students to see the value of science, since previous studies in the cognition sciences have shown that value attribution for a given activity is also related to the understanding of it. As Kruger and Dunning (1999) stated: “the skills that engender competence in a particular domain are often the very same skills necessary to evaluate competence in that domain—one’s own or anyone else’s” (p. 1121). This recognition of the value of science and the understanding of its methods and processes as sources of knowledge are, indeed, among the foundations of scientific literacy (Hodson, 2014).

From the perspective of pedagogical practices, a focus on EE continuum allows more questions to emerge, both by questioning the nature of the data and its multiple possibilities for transformation and by reflecting on the nature of the explanations. Regarding explanations, there are different levels of complexity and sophistication (see Machamer et al., 2000); however, as we can also see in the previous case study, these levels are also associated with a real understanding of what is evidence and what distinguishes it from the data. Epistemological conversations about data-texts transformations have the potential to foment situations in which students can genuinely exercise an epistemic agency, going beyond an individual sense construction that is often insufficient to understand the construction of the evidence.

Epistemological conversations demand an active teaching action capable of promoting student engagement. The development and mediation of the meaning by the teacher as facilitator depend on the way the dialog is conducted. The teacher is responsible for introducing and supporting the orientation and evaluation of the dialogs that are established throughout the process of building student autonomy. Such a path, which can be characterized by what Stroupe (2014) called “classrooms as a science practice community” (p. 489), is prompted by learning events in dialogs that occur with the approach of science as practice. For Stroupe (2014), the negotiation of what matters or does not count as knowledge (which is developed along the EE continuum, for example) is a fundamental dimension for the redefinition of students’ roles and for the construction of an epistemic agency. In such a context, we understand that the EE continuum can help teachers in this journey, as a discussion about data-texts transformations allows students not only to continuously express their thoughts but also to deal with different forms of scientific practices and representations (Sandoval et al., 2000).

Accordingly, the shift to the EE continuum that we are advocating here is also in tune with Osborne’s (2018) argument that the difficulty in placing scientific reasoning at the center of science curricula is the lack of clarity of its role in learning sciences. We agree with Osborne that scientific reasoning is a highly complex skill which requires a combination of purposeful strategies to be developed. In this context and considering what we presented so far, we argue that focusing on the EE continuum can both contribute to a less distorted view of scientific processes and act as a substrate for teaching the various modes of scientific reasoning, mainly as a result of an epistemic agency that places students as data producers instead of data collectors (Hardy et al., 2020).

Each transformation that takes place along the EE continuum represents an opportunity for epistemic discourse. The successive decisions made along with the data-texts transformations thus constitute powerful didactic possibilities to explore the epistemic transformations that occur along with the production of knowledge. Identifying, organizing, and evaluating what counts as scientific questions, measurements, observations, data, patterns, models, and explanations are actions that can be decided collectively between students and teachers throughout planned learning progressions.

In the planning dimension, teachers can envisage which are the most relevant transformations among the EE continuum, considering the learning goals and the abilities to be developed. Such a framework might be a didactic guideline, allowing the teacher to navigate in different directions (in EE continuum) for planning and conducting investigative activities. In the context of inquiry and learning progressions, it is possible to take into account that the data-texts transformations can occur over several classes and that the inquiry process is dependent on what is being investigated (Hodson, 2014). It is still worth pointing out that, along EE continuum and epistemological conversations, a series of epistemic practices are mobilized. This can contribute to the development of student autonomy, either through the construction and use of inscriptions (Prain & Tytler, 2012) or through constant communication that involves the social practices of science.

This paper contributes to recent dialogs and research on scientific reasoning. Our theoretical framework endorses the inquiry perspective, highlighting the importance of the EE continuum for planning and monitoring learning progressions with particular attention to the development of well-informed views of the idea of evidence. In this context, we believe that the use of the EE continuum may act as a form of scaffolding for learners. In our classroom-based example, we tried to demonstrate not only that students and teachers can engage in epistemological conversations involving data-texts transformations, but mainly how the EE continuum can be a valuable tool in curriculum design.

The approach we propose in this paper is also in dialog with the emerging concerns of helping students to navigate through the scientific information available in the mainstream media. The current COVID-19 pandemic has exposed citizens to a range of scientific inscriptions and representations, carrying with them debates about how data-texts are created and interpreted. We now routinely witness the mainstream media discussing (with professional scientists, science enthusiasts, or opinion holders not necessarily based on science), for example, sample sizes, sampling strategies, what counts as data, and what is or is not scientific evidence. These questionings prompt reflections on data-texts transformations, especially as they are also followed by questions on how scientific information is communicated. In addition, in the post-truth era, science’s delegitimization in a pandemic world has shown us the need to deal with data-texts transformations more effectively in the context of science education.

As such, our position highlights the value of the EE continuum as an intentional pedagogical tool for analyzing student engagement and development, identifying and leveraging key points of discussion, developing scaffoldings (e.g., Sandoval & Reiser, 2004), and informing curriculum development. Future derivative research includes looking at teachers’ use of the framework when planning and implementing lesson sequences. We hope that our ideas and thinking will provide a platform for (a) reasoning about the epistemological processes involved in the construction of scientific knowledge; and (b) fostering discussions about the epistemological *conversations* of doing science within educational contexts. A shift of emphasis on the epistemic processes within science is important for two main reasons: it has implications for the design of contemporary learning environments, and it has much to contribute in our understanding of the nature and processes of scientific practice. Furthermore, considerations for increasing the focus on data and evidence in contexts such as policy making and environmental decision-making enable data-text conversations to be another opportunity for linking school science with the sociocultural contexts of students’ daily lives that necessitate different types and levels of individual decision-making.
